# Clinical Characteristics and Outcomes of Pediatric Patients With Immune Thrombocytopenic Purpura in King Abdulaziz Medical City and King Abdullah Specialist Children’s Hospital: A 10-Year Study

**DOI:** 10.7759/cureus.11366

**Published:** 2020-11-06

**Authors:** Khalid W Alwadi, Ali Alomari, Abdulmohsen K Alrugaib, Abdulrahman Alrubayea, Musab Alzoman, Fahad Alkahtani

**Affiliations:** 1 Medicine, King Saud Bin Abdulaziz University for Health Sciences, King Abdulaziz Medical City, Ministry of National Guard - Health Affairs, Riyadh, SAU; 2 Pediatric Hematology/Oncology, King Abdulaziz Medical City, Ministry of National Guard - Health Affairs, Riyadh, SAU; 3 Medicine, King Saud Bin Abdulaziz University for Health Sciences College of Medicine, Riyadh, SAU

**Keywords:** itp in pediatric, itp, immune thrombocytopenic purpura, idiopathic thrombocytopenic purpura, immune thrombocytopenia

## Abstract

Background

Immune thrombocytopenic purpura (ITP) can be defined as “an immune-mediated acquired disease characterized by a transient or persistent decrease in the platelet count”. Medical treatment is usually not needed but, in some cases, intravenous immunoglobulin G (IVIG), corticosteroids, and anti-D immunoglobulins are used. Splenectomy can be an option for chronic cases with no response to pharmacological treatments. The aim of this study was to describe the clinical characteristics and outcomes of pediatric patients with ITP in King Abdulaziz Medical City (KAMC) and King Abdullah Specialist Children’s Hospital (KASCH) in a 10-year period.

Methods

The study was conducted at KAMC and KASCH. The number of recorded cases was 95, which included all ITP patients aged 1 to 14 from both genders who presented to KAMC previously and KASCH currently from January 1, 2007, to December 31, 2017. The data analysis and entry were performed using the Statistical Package for the Social Sciences (SPSS) version 25 (IBM Corp., Armonk, NY).

Results

Among 95 pediatric patients with ITP, 51 (53.7%) were males and 44 (46.3%) were females, with a median age of 4.00 ± 3.977. Among them, 84 (92.7%) had purpura, 38 (46.3%) had epistaxis, 43 (39%) had petechia, 17 (24.3%) had fever, and 20 (19.5%) had gum bleeding. Out of 95 patients, 91 (95.8%) were given treatment. Out of those 91 patients who were given treatment, IVIG was used in 84 (92.3%), steroids were used in 44 (48.4%), 14 patients received platelet transfusion (15.4%), rituximab was used in 7 (7.7%), and splenectomy was done in 5 (5.5%); 32 (33.7%) cases were considered chronic (more than one year), and 63 (66.3%) were considered acute. Among chronic patients, only one death was recorded, while in acute, no deaths were recorded.

Conclusion

In conclusion, ITP is an autoimmune disease that decreases platelet count. The results showed a significant difference in treatment compared to the literature but similar results in other aspects.

## Introduction

Immune thrombocytopenic purpura (ITP) can be defined as “an immune-mediated acquired disease of adults and children characterized by transient or persistent decrease in the platelet count and, depending upon the degree of thrombocytopenia, increased risk of bleeding” [1]. It is characterized as acute if it resolves within one year and chronic if it persists for more than one year [2]. In children, ITP usually presents with symptoms and signs of thrombocytopenia such as gum bleeding, nose bleeding (epistaxis), easy bruising, purpura, and intracranial hemorrhage. As for the lab findings, the platelet count is less than 100×10^9^/L. Symptoms usually appear if the platelet count drops below 20×10^9^/L. Usually, the lower the platelet count is, the more severe the condition is [3-5]. ITP is diagnosed after excluding other causes of thrombocytopenia.

Regarding treatment, children normally do not require medical intervention. However, if the platelet count level is less than 20×10^9^/L [1], medical intervention is required to maintain an adequate platelet count to prevent serious bleeding. This is usually done through intravenous immunoglobulin G (IVIG), corticosteroids, anti-D immunoglobulins, and other treatment modalities [6]. For chronic cases with no response to pharmacological treatments, splenectomy is an option [7,8]. Patients are considered in remission if the platelet count doesn’t drop below 100×10^9^/L after the cessation of treatment. Epidemiologically, according to the literature, the incidence rate is higher in males than females. The outcome for children is usually acute, whereas adults usually have a chronic outcome [9-11].

There is a paucity of recent data on ITP in children in Saudi Arabia, which this research aimed to compensate for [12]. Thus, all pediatric ITP patients received in King Abdulaziz Medical City (KAMC) in the last 10 years were reviewed while comparing KAMC experience and other tertiary hospitals worldwide.

## Materials and methods

The study was conducted in the Pediatric Hematology/Oncology department at KAMC and King Abdullah Specialist Children’s Hospital (KASCH), a tertiary hospital in Riyadh. The number of recorded cases was 95, which included all ITP Saudi and non-Saudi patients of both genders aged 1 to 14 years from January 1, 2007, to December 31, 2017, who presented to KAMC previously and KASCH currently, as KASCH was established in 2015 and all pediatric patients in National Guard - Health Affairs in Riyadh were transferred to KASCH. Any patient who presented with thrombocytopenia that can be explained by other conditions was excluded. The study is a retrospective case series using a data collection form, which was based on a study of other data collection forms around the hospital. The data collection form was appraised through a pilot study, which included 12 cases.

Data entry was done through Microsoft Excel. Data analysis was performed using the Statistical Package for the Social Sciences (SPSS) version 25 (IBM Corp., Armonk, NY). Categorical data (symptoms, gender, outcome, etc.) was presented using bar charts, frequencies, and percentages. As for the numerical data, it was presented in means and standard deviations. Statistical tests were not used as the study is focused on providing statistics about the presentation of the disease, treatment used in the hospital, and the outcome of the disease.

## Results

Demographics

In this retrospective study, 95 medical records of pediatric patients aged 1 to 14 with ITP were evaluated. They were categorized into three age groups (1-5, 6-10, and 11-14 years of age).

Among 95 pediatric patients with ITP, 51 (53.7%) were males and 44 (46.3%) were females, with a median age of 4.00 ± 3.977. In the three age categories, 60 (63.2%) were between 1 and 5 years of age, 16 (16.8%) were between 6 and 10 years of age, and 19 (20%) were between 11 and 14 years of age. As for the ratio of Saudis to non-Saudis, 90 (94.7%) were Saudis and 5 (5.3%) were non-Saudis (Table 1).

**Table 1:** Patient demographics

	n	Percentage
Age group (years)		
1-5	60	63.2%
6-10	16	16.8%
11-14	19	20%
Gender		
Male	51	53.7%
Female	44	46.3%
Nationality		
Saudi	90	94.7%
Non-Saudi	5	5.3%

Clinical characteristics

**Figure 1:**
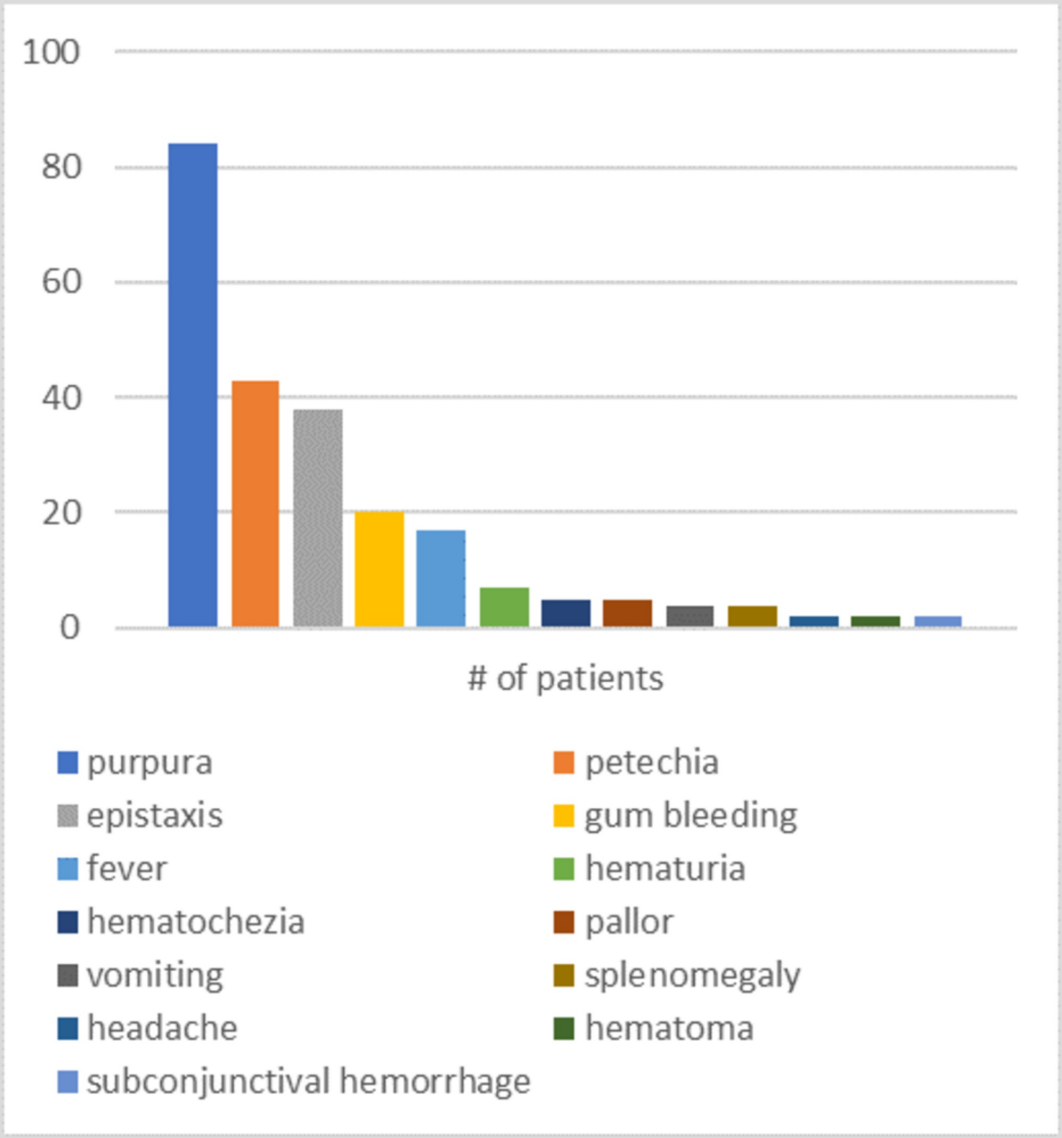
Signs and symptoms observed

Out of 95 patients, 84 (88.4%) patients had purpura, making it the most common sign, 43 (45.3%) patients had petechia, 38 (40%) had epistaxis, 20 (21.1%) had gum bleeding, 17 (17.9%) had fever, 7 (7.4%) had hematuria, 5 (5.3%) had hematochezia, 5 (5.3%) had pallor, 4 (4.2%) had vomiting, 4 (4.2%) had splenomegaly, 2 (2.1%) had headache, 2 (2.1%) had hematomas, 2 (2.1%) had subconjunctival hemorrhage, 1 (1.1%) patient had subarachnoid hemorrhage, 1 (1.1%) had abdominal tenderness, 1 (1.1%) had hemorrhagic gastropathy, 1 (1.1%) had hepatomegaly, 1 (1.1%) patient had weight loss, 1 (1.1%) had muscle pain, 1 (1.1%) had blurred vision, and 1 (1.1%) patient was asymptomatic (Figure 1). As for the platelet count, the study demonstrated a mean platelet count of 17.2×10^9^/L on admission (SD ±29.66395).

Treatment

**Figure 2:**
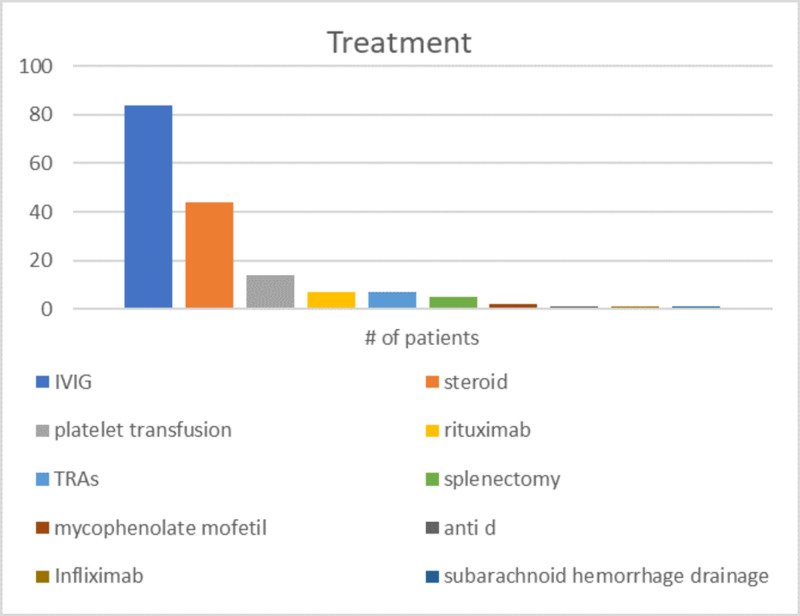
Treatment modalities used IVIG, intravenous immunoglobulin G; TRA, thrombopoietin receptor agonist

Out of 95 patients, 91 (95.8%) patients were given treatment and 4 (4.2%) only had watchful observation. Among those who were treated, IVIG was the commonest option as it was used in 84 (92.3%) patients due to it being the first line of treatment in the hospital’s treatment protocol (Figure 2). Steroids were used in 44 (48.4%) patients, 14 (15.4%) patients received platelet transfusion, rituximab was used in 7 (7.7%) patients, eltrombopag and romiplostim were used in 7 (7.7%), and splenectomy was done in 5 (5.5%). Other modalities were mycophenolate mofetil (MMF) in two (2.2%) patients, anti-D in one (1.1%) patient, and infliximab used in one (1.1%) patient. Also, one (1.1%) patient had subarachnoid hemorrhage drainage.

Outcome

A total of 63 (66.3%) cases were considered acute, while 32 (33.7%) were considered chronic (more than one year). As for their outcome, all acute patients went into remission, and while out of 32 chronic patients, seven were alive in remission, 24 were still not resolved as of June 2019, and one patient died.

## Discussion

In this retrospective study, multiple clinical features of pediatric ITP patients were identified. After that, results were compared with multiple similar local, regional, and international studies. Hence, the comparisons were focused on the following points: demographical data, clinical characteristics, and outcomes.

Demographics

This study differed from other studies in gender distribution. Locally and regionally, it showed an almost equal distribution of genders in contrast to male predominance in a study by Al-Mulla et al. in Qatar (62% male) [12] and in a study by Al Fawaz in King Khalid University Hospital (61% male) [13], which contrasts to the female predominance seen in Abha (41% male) [14]. Internationally, there was male predominance in a study by Grainger et al. in UK hospitals (57% male) [15], while Zeller et al. in Norway [16] showed female predominance (43% male). On the other hand, Glanz et al. in Colorado [17] - similar to this study - showed an equal distribution of genders. As for the age distribution, this study was similar to those in Iraq, Qatar, and King Khalid University Hospital [11-13].

Clinical characteristics

Only one of the patients came without a manifestation of the disease’s signs and symptoms. Nearly 75% of patients had a platelet count under 20×10^9^/L, which is slightly less than that in an international study by Kühne et al. [2], Iraq’s study [11] and Abha’s [14], where it was around 80%. On the other hand, only 68% of patients from the study by Al-Mulla et al. in Qatar [12] had a below 20×10^9^/L count. Additionally, the most common presentation in the patients was purpura or petechia followed by epistaxis, which goes along with studies by Kühne et al. internationally [2], Al-Zuhairy in Iraq [11], Al-Mulla et al. in Qatar [12], and Al Fawaz in King Khalid University Hospital [13].

Treatment

In this study, almost all patients (95.8%) received treatment that is similar to the study in Iraq [11] but different from multiple other studies. In King Khalid University Hospital, 75% of patients received treatment [13], and in the international study by Kühne et al., 69% of patients received treatment [2], while in the UK, it was as low as 16% [15]. The high treatment rate may be attributed to the fact that almost all the patients who presented to the hospital were symptomatic except for one.

Outcome

Similar to multiple local, regional, and international studies, the results have shown a predominantly acute pattern of the disease (66.3%). Starting locally, in Abha [14], 65.2% of patients were acute while King Khalid University Hospital’s patients were 55.1% acute [13]. Regionally, in Al-Zuhairy’s study in Iraq, 72% patients were acute [11], while in the study by Al-Mulla et al. in Qatar [12], 62% of cases were acute. Internationally, 52.7% of pediatric patients in Kühne et al.’s study - a multicenter international study - were acute cases [2].

## Conclusions

In summary, ITP is an autoimmune disease characterized by a decreased platelet count and bleeding tendency. Compared with previous local, regional, and international studies, the present study showed similar results to the literature except for treatment. In treatment, most of the patients were given medical treatment, while other studies used watchful waiting more.

## References

[1] Rodeghiero F, Stasi R, Gernsheimer T (2009). Standardization of terminology, definitions and outcome criteria in immune thrombocytopenic purpura of adults and children: report from an international working group. Blood.

[2] Kühne T, Buchanan G, Zimmerman S (2003). A prospective comparative study of 2540 infants and children with newly diagnosed idiopathic thrombocytopenic purpura (ITP) from the Intercontinental Childhood ITP Study Group. J Pediatr.

[3] Bansal D, Rajendran A, Singhi S (2014). Newly diagnosed immune thrombocytopenia: update on diagnosis and management. Indian J Pediatr.

[4] Saeidi S, Jaseb K, Asnafi AA (2014). Immune thrombocytopenic purpura in children and adults: a comparative retrospective study in Iran. Int J Hematol Oncol Stem Cell Res.

[5] Mushtaq N, Alam MM, Fadoo Z (2014). Idiopathic thrombocytopenic purpura in children: a 10 years experience at tertiary care hospital. J Pak Med Assoc.

[6] Neunert C, Lim W, Crowther M, Cohen A, Solberg L, Crowther MA (2011). The American Society of Hematology 2011 evidence-based practice guideline for immune thrombocytopenia. Blood.

[7] Kumar P, Clark M (2016). Kumar and Clark's Clinical Medicine, Ninth Edition. https://www.elsevier.com/books/kumar-and-clarks-clinical-medicine/kumar/978-0-7020-6601-6.

[8] Aleem A (2010). Durability and factors associated with long term response after splenectomy for primary immune thrombocytopenia (ITP) and outcome of relapsed or refractory patients. Platelets.

[9] Grimaldi-Bensouda L, Nordon C, Leblanc T (2017). Childhood immune thrombocytopenia: a nationwide cohort study on condition management and outcomes. Pediatr Blood Cancer.

[10] Shirahata A, Fujisawa K, Ishii E (2009). A nationwide survey of newly diagnosed childhood idiopathic thrombocytopenic purpura in Japan. J Pediatr Hematol Oncol.

[11] Al-Zuhairy S (2013). Evaluation of prognostic factors in newly diagnosed childhood primary immune thrombocytopenia (ITP): two-year prospective study at Al-Sadder Hospital, Missan Province. Med J Babylon.

[12] Al-Mulla N, Bener A, Amer A, Abu Laban M (2009). Idiopathic thrombocytopenic purpura in childhood: a population-based study in Qatar. (Article in Portuguese). J Pediatr (Rio J).

[13] Al Fawaz I (1993). Childhood idiopathic thrombocytopenic purpura: experience at King Khalid University Hospital, Riyadh. Ann Saudi Med.

[14] Al-Suheel A, Shati A, Almedhesh SA, Alshehri DB, Alzahrani M (2014). Immune thrombocytopenia among children living at a high altitude region: a hospital-based retrospective study. Med J Cairo Univ.

[15] Grainger JD, Rees JL, Reeves M, Bolton-Maggs PH (2012). Changing trends in the UK management of childhood ITP. Arch Dis Child.

[16] Zeller B, Helgestad J, Hellebostad M, Kolmannskog S, Nystad T, Stensvold K, Wesenberg F (2000). Immune thrombocytopenic purpura in childhood in Norway: a prospective, population-based registration. Pediatr Hematol Oncol.

[17] Glanz J, France E, Xu S, Hayes T, Hambidge S (2008). A population-based, multisite cohort study of the predictors of chronic idiopathic thrombocytopenic purpura in children. Pediatrics.

